# Single-layer graphene oxide film grown on α-Al_2_O_3_(0001) for use as an adsorbent

**DOI:** 10.3762/bjnano.16.79

**Published:** 2025-07-10

**Authors:** Shiro Entani, Mitsunori Honda, Masaru Takizawa, Makoto Kohda

**Affiliations:** 1 Quantum Materials and Applications Research Center, National Institutes for Quantum Science and Technology, Takasaki, Gunma 370-1292, Japan; 2 Honda’s Lab for Development of Future Clay Materials Research, Japan Atomic Energy Agency, Tokai, Ibaraki 319-1195, Japan; 3 College of Science and Engineering, Ritsumeikan University, Kusatsu, Shiga 525-8577, Japan; 4 School of Engineering, Tohoku University, Aoba-ku, Sendai 980-8579, Japan

**Keywords:** cesium adsorption, chemical vapor deposition, electronic state analysis, graphene oxide, X-ray absorption fine structure

## Abstract

Graphene oxide (GO) is expected to be one of the most promising adsorbents for metal ions, including radioactive nuclides in aqueous solutions. Large-area and single-layer graphene oxide (SLGO) grown on α-Al_2_O_3_(0001) was used as a model structure of GO since the aggregation and re-stacking of the GO sheets prevent the adequate analysis of the adsorption state. The SLGO film was obtained by oxidizing monolayer graphene grown by metal-free chemical vapor deposition on the α-Al_2_O_3_(0001) surface, and the adsorption state was determined by surface analytical techniques. It was clarified that Cs adsorbs on oxygen functional groups by substituting with H atoms from carboxyl and hydroxy groups. It is also estimated that the weight adsorption capacity of SLGO in the 1.0 mol/L-Cs aqueous solution is as much as approximately 70 wt %. It has been demonstrated that GO has great potential to be a promising adsorbent for Cs in aqueous solutions.

## Introduction

Graphene oxide (GO) is oxidized graphene and its surface and periphery are partially modified by epoxy, hydroxy, and carboxy functional groups [1–2]. GO can be thinned to a monolayer of one carbon atom and has a high level of water affinity. Consequently, GO can be expected to have a wide range of applications, such as primers, thermally conductive materials, transparent electrodes, and adsorbent materials [3–13]. GO is typically synthesized by oxidizing graphite. Several methods have been reported, including the Broadie and Hummers methods, contingent upon the oxidizing agent employed [8,14–15]. GO possesses the ultimate large surface area, low-cost production, and high chemical stability. Therefore, GO has a potential prospect to be an efficient adsorbent for metal ions from aqueous solutions. In order to realize its application use, it is necessary to form large-area GO films with well-controlled thickness. However, the conventional methods for the fabrication of such tailored GO films present considerable challenges. Conventionally, GO films are obtained by a drop-casting technique, wherein GO flakes dispersed in a solvent are cast onto a substrate [16–17]. This technique does not allow for the control of number of layers.

Consequently, studies have been conducted to synthesize large-area and single-layer GO (SLGO) films. As mentioned above, GO films have typically been fabricated through casting small pieces of GO flakes onto a substrate. In this study, the SLGO film was synthesized by oxidizing single-layer graphene (SLG) grown by metal-free chemical vapor deposition (CVD) on a α-Al_2_O_3_(0001) substrate. The strong interface interaction between SLG and α-Al_2_O_3_(0001) can minimize peeling off of SLG from the α-Al_2_O_3_(0001) substrate following the oxidation process [18]. This allows us to obtain a large-area SLGO film, which can then be subjected to evaluation of the adsorption properties.

Subsequent to the synthesis of SLGO/α-Al_2_O_3_(0001), further studies were conducted into its adsorption properties. A significant accident at a nuclear facility can result in the release of substantial amounts of radioactive cesium (Cs) and strontium (Sr) into the environment [19–20]. The development of a method for reducing the volume of these radioactive waste is imperative. Cs and Sr exhibit strong adsorption to particles of rocks and soils, resulting in their water insolubility and substantial volume. A viable method for the disposal of radioactive waste is the application of an acid treatment, which has been demonstrated to be effective in the efficient separation of radioactive Cs contaminants from rocks and soils [21]. The resulting solution is a substantial quantity of low-level radioactive treated water. Therefore, the adsorbent needs to possesses high-adsorption performance, a rapid reaction rate, and cost-effectiveness in production. Romanchuk et al. have demonstrated that GO is effective in the removal of actinides from nuclear wastewaters [8]. However, the adsorbing mechanism of metal ions to the GO surface, such as adsorption sites, remains to be elucidated. It has been demonstrated that GO tends to aggregate during the process of metal ion adsorption [8], which complicates the evaluation of GO using surface analytical techniques. The SLGO film synthesized in this study is adhered to the α-Al_2_O_3_(0001) surface, which prevents GO aggregation and multilayer stacking. It is expected that this will enable detailed evaluation of Cs adsorption on GO.

In this study, the SLGO surface after Cs adsorption was analyzed by surface analytical tools. This enabled us to elucidate the adsorbing sites and electronic state of Cs on SLGO. Additionally, we examined the electronic structure of Cs adsorbed on SLGO in several different solutions with pH values of 4, 7, and 9. Both the electronic structure and the normalized amount of Cs adsorbates were dependent on the pH scale. These fundamental aspects provide us important information for developing new adsorbent materials using GO.

## Results and Discussion

### Large-area and single-layer graphene oxide growth

**Figure 1:**
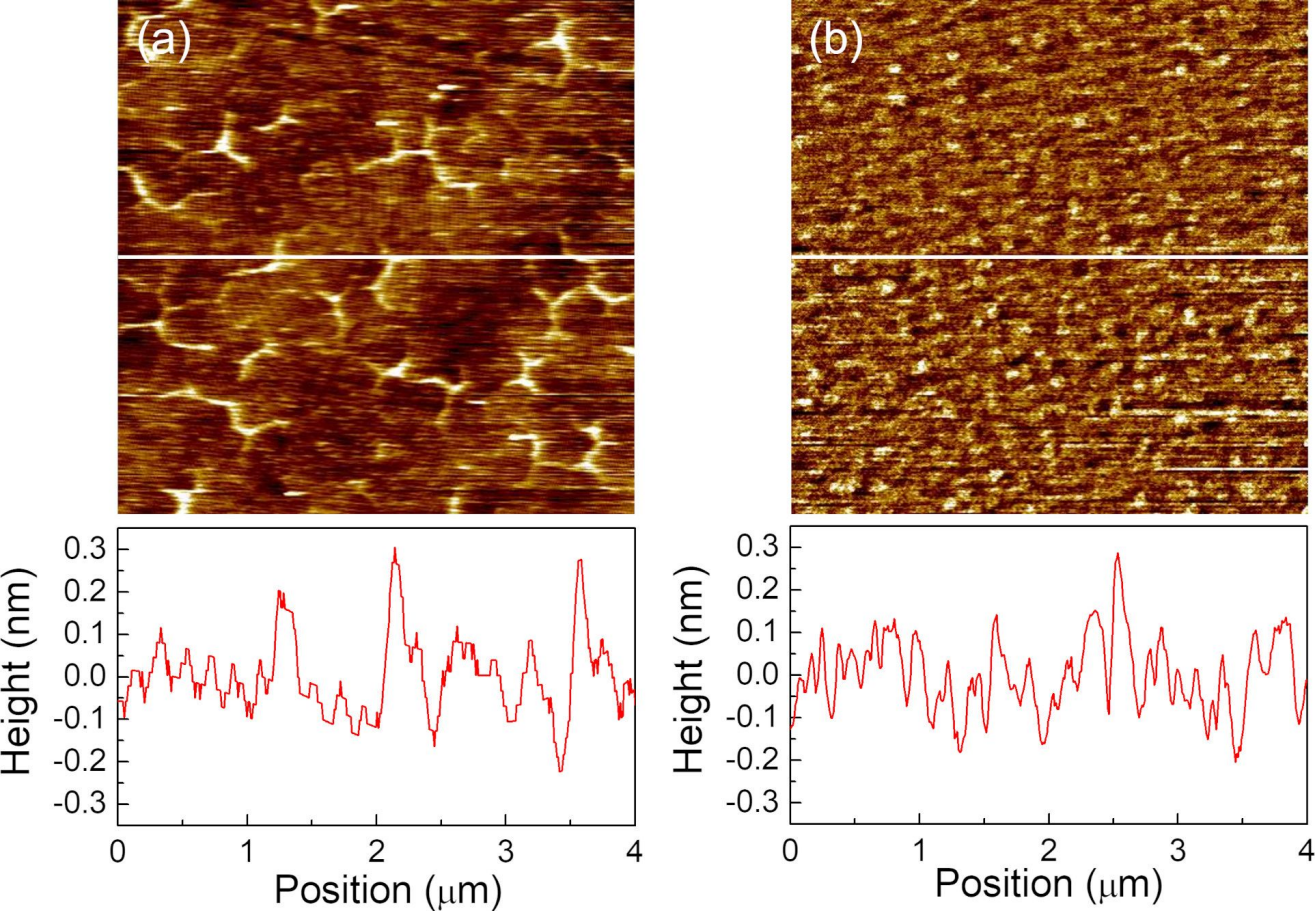
AFM images of (a) SLG/α-Al_2_O_3_(0001) and (b) SLGO/α-Al_2_O_3_(0001).

**Figure 2:**
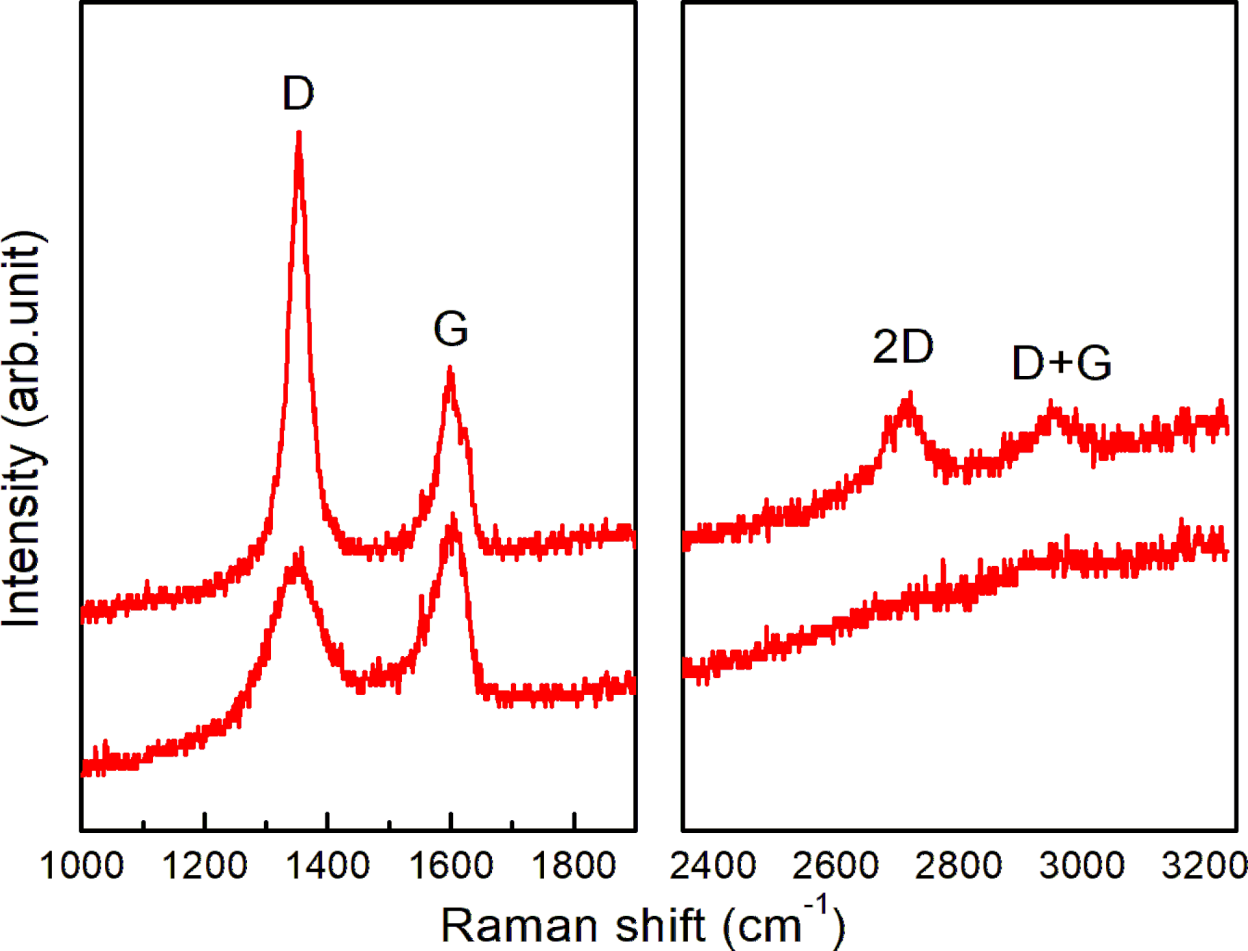
Two sets of Raman spectra from SLG/α-Al_2_O_3_(0001) (upper) and SLGO/α-Al_2_O_3_(0001) (lower).

Figure 1 shows an atomic force microscopy (AFM) image of SLG and SLGO on α-Al_2_O_3_(0001) substrates. The as-grown SLG film has an atomically flat surface and wrinkles with its height less than 0.4 nm [18]. The single layer of graphene was confirmed through X-ray photoelectron spectroscopy (XPS) peak intensity analysis and profiles of normal-incidence X-ray standing wave (NIXSW) spectroscopy [18]. In the SLGO film, the wrinkles disappeared and the surface roughness increased. The root mean square surface roughness (RMS) of the SLGO film is estimated to be less than 0.13 nm. The changes of the local structure are confirmed by Raman spectroscopy measurements. Figure 2 shows two sets of the Raman spectra of SLG and SLGO. In SLG/α-Al_2_O_3_(0001), four prominent peaks are identified, which are assigned to the D band (around 1355 cm^−1^), G band (around 1585 cm^−1^), 2D band (around 2700 cm^−1^), and D+G band (around 2900 cm^−1^). The presence of intense D and D+G peaks is indicative of the existence of graphene film disorder. This phenomenon can be attributed to the reduced size of the graphene grains that are produced at lower growth temperatures [18]. Following the oxidation process, a broadening of all peaks is observed, accompanied by a substantial decrease in the intensity of the 2D band. These alterations are attributed to the presence of defects and a decline in crystallinity, which is associated with the attachment of oxygen functional groups. These spectral features were consistent with the findings reported in [22].

**Figure 3:**
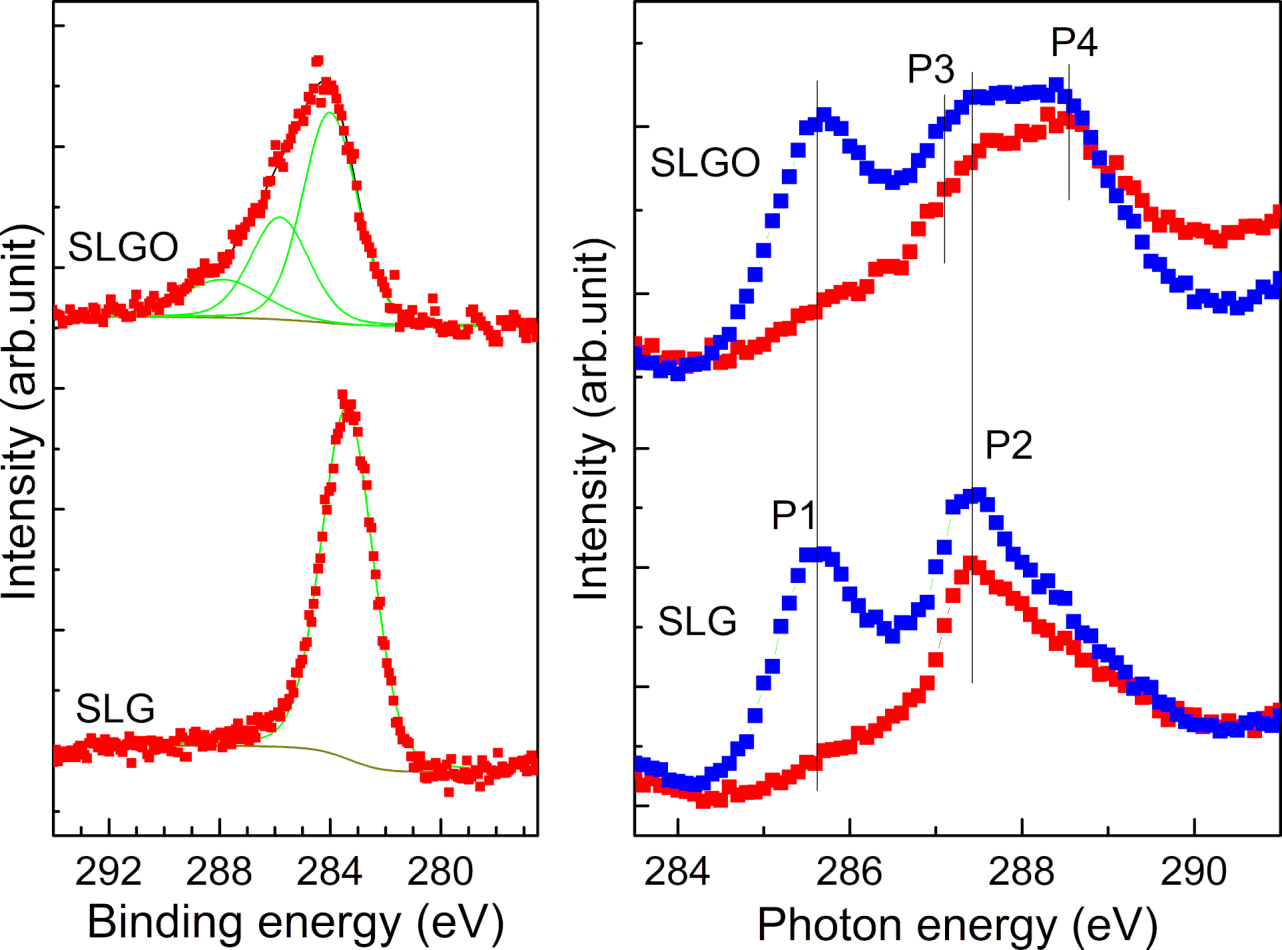
(a) C 1s core level XPS and (b) polarization dependence of C K-edge NEXAFS spectra of SLG/α-Al_2_O_3_(0001) (lower) and SLGO/α-Al_2_O_3_(0001) (upper) The incident angles of photon beams from the surface are 90° (red) and 30° (blue), respectively.

At the same time, the significant changes arose after the oxidization of SLG in the C 1s core XPS and C K-edge near-edge X-ray absorption fine structure (NEXAFS) spectra. Figure 3a shows C 1s XPS spectra of SLG and SLGO. In SLG, an intense peak is observed at 283.4 eV. The C 1s XPS spectra of SLG/α-Al_2_O_3_(0001) is shifted to the lower binding energies by 1.0 eV compared with that of graphite (284.4 eV) [23]. This is due to p-type doping of SLG associated with a strong electrostatic interaction between SLG and the α-Al_2_O_3_(0001) at the interface [18]. In SLGO, on the other hand, a broad structure arose in the higher binding energy region of the intense peak. The structure is originated from the introduction of the oxygen functional groups such as hydroxy and carbonyl groups [24]. It can be reasonably considered that the introduction of the local structures in SLGO causes the relaxation of the interfacial strains and the disappearance of wrinkles as seen in Figure 1b. Figure 3b shows the C K-edge NEXAFS spectra of SLG and SLGO. Two peaks P1 and P2 are observed at 285.4 eV and 287.5 eV. These peaks are assigned to the C 1s to π*(C=C) and to σ*(C–H) transitions, respectively [25]. The intensity of P1 at the grazing incidence is much larger than that at the normal incidence. This indicates that the graphene sheet is parallel to the substrate surface. Two additional small features arose in SLGO; 287.1 eV (P3) and 288.4 eV (P4). These are assigned to C 1s to π*(C–OH) and to σ*(C=O) transitions, respectively [26–28]. It is also found that P3 and P4 show no X-ray incident angle dependence, which suggests that oxygen functional groups attached to graphene have no particular orientation distribution.

### Cs adsorption

**Figure 4:**
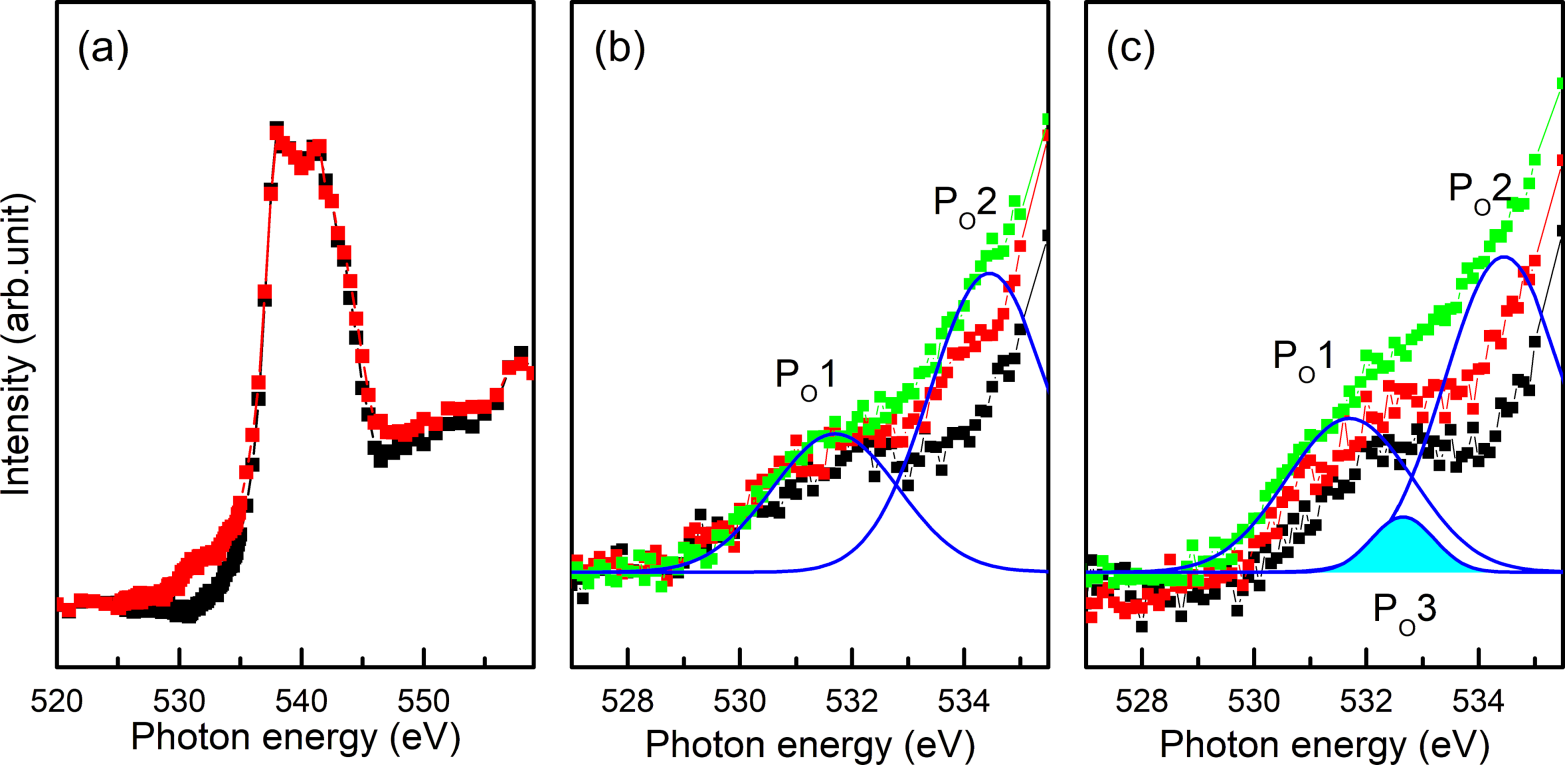
(a) O K-edge NEXAFS of α-Al_2_O_3_(0001) (black) and SLGO/α-Al_2_O_3_(0001) (red). (b) and (c) Polarization dependence of O K-edge NEXAFS of SLGO/α-Al_2_O_3_(0001) and Cs-adsorbed SLGO/α-Al_2_O_3_(0001), respectively. The incident angles of the photon beams from the surface are 90° (black), 55° (red), and 30° (green), respectively.

The electronic structure of Cs-adsorbed SLGO was also observed by employing NEXAFS. Figure 4a shows the O K-edge NEXAFS spectra of the α-Al_2_O_3_(0001) substrate and SLGO/α-Al_2_O_3_(0001). A pronounced structure is observed at around 540 eV. This structure is originated from the Al–O in the α-Al_2_O_3_(0001) substrate [29–30]. After the SLGO growth, a small shoulder arises in the lower photon energies. Spectral analysis of this structure shown in Figure 4b indicates that the shoulder consists of two components; P_O_1 (531.6 eV) and P_O_2 (534.4 eV). These are assigned to π*(O=C from the carboxyl groups) and σ*(O–H from the hydroxyl groups), respectively [23]. It is found that the intensity of P_O_2 shows an incident angle dependence (i.e., the peak intensity measured at the grazing incidence is slightly larger than that at the normal incidence). This indicates that the O–H bond is oriented roughly along the normal direction to the substrate. In the O K-edge spectrum of Cs-adsorbed SLGO shown in Figure 4c, a new component appears at the photon energies between P_O_1 and P_O_2; P_O_3 (532.7 eV). As detailed in the following section, Cs has been found to be adsorbed on oxygen functional groups through the process of ion exchange. Consequently, it can be inferred that P_O_3 is associated with the O–Cs bonds. It should be noted that the intensity of P_O_3 measured at grazing incidence is slightly larger than that at normal incidence. This indicates that the O–Cs bond tends to be oriented along the normal direction to the SLGO sheet.

**Figure 5:**
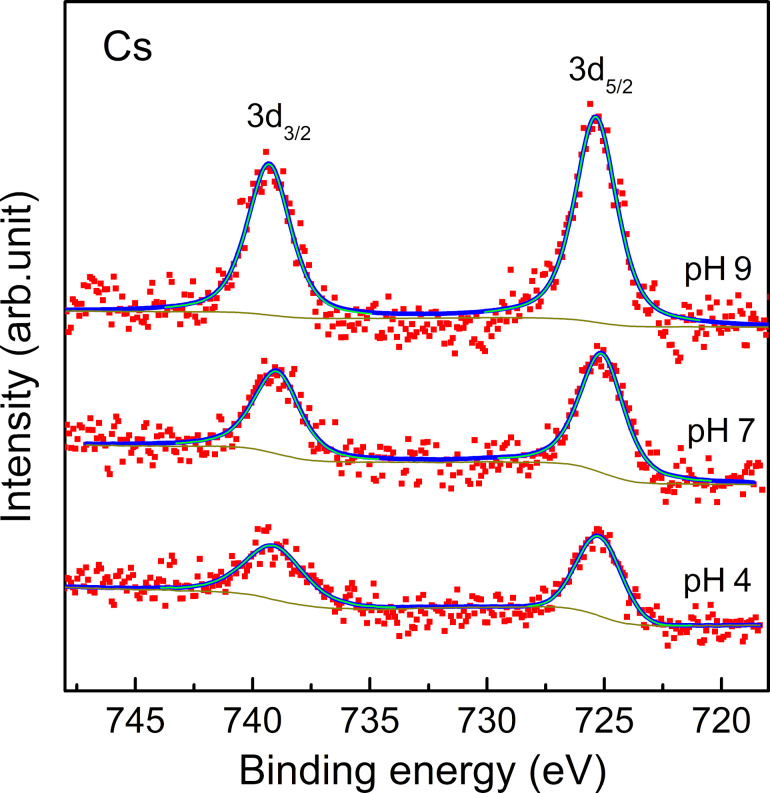
Cs 3d core level XPS spectra of Cs-adsorbed SLGO from various CsCl solutions with different pH values (pH 4, 7, and 9).

For the purpose of elucidating the adsorbing mechanism of the Cs atoms to the SLGO sheet, we investigated the electronic structure and chemical properties of adsorbed Cs by changing the pH level of the Cs aqueous solutions. Figure 5 shows Cs 3d XPS spectra of Cs-adsorbed SLGO under three different pH values. No energy shift was observed among these spectra. This indicates that the chemical state of adsorbed Cs is identical regardless of the pH value. In contrast, it should be noted that the capacity of the Cs adsorption significantly increases with an increasing pH value. It has been reported that the oxygen functional groups in GO have dissociation constants p*K* = 4.3, 6.6, and 9.8 [31]. It can be considered that Cs adsorbs on the oxygen functional groups through a mechanism of ion exchange. This process involves the substitution of H atoms from oxygen functional groups, such as carboxyl and hydroxy groups, with Cs atoms. It can therefore be surmised that the ability of SLGO for the Cs adsorption is significantly decreased in strong acid aqueous solutions due to the suppression of hydrogen dissociation from oxygen functional groups in SLGO. It is claimed that the pH value of the aqueous solution should be maintained at neutrality and/or alkalinity for the efficient Cs adsorption by SLGO. With regard to the quantitative analysis, it is also noted that SLGO shows high adsorption capacity of Cs. That is, the weight adsorption capacity in the CsCl solution with pH 7 is estimated to be 650–850 mg Cs per 1 g SLGO, which corresponds to 70 wt %, from the XPS analysis based on the peak intensity ratio between C 1s and Cs 3d. This value is considerably higher than that of existing adsorbents, including zeolite (189 mg·g^−1^) [32–34]. GO is comprised of light elements, which results in a significantly high calculated adsorption capacity.

## Conclusion

In this study, the synthesis of the large-area SLGO film was accomplished through the oxidation of CVD-grown SLG/α-Al_2_O_3_(0001). We found that the change in the electronic state from graphene to GO is attributed to oxidation. This was accompanied by the decrease of the π*(C=C) state and the appearance of states derived from oxygen functional groups. Subsequently, the adsorption mechanism of Cs on GO was investigated using SLGO/α-Al_2_O_3_(0001) as the model structure. It was found that Cs adsorbs on SLGO by the substitution of H from oxygen functional groups, such as carboxyl and hydroxy groups. It was also indicated that SLGO shows the high Cs adsorption capacity of 650–850 mg·g^−1^ in the 1.0 mol/L-Cs aqueous solution. SLGO adsorbs Cs via hydrogen exchange, resulting in an accelerated adsorption rate and a higher weight adsorption capacity compared to existing adsorbents, including zeolites. Consequently, SLGO was demonstrated to be a promising adsorbent. As SLGO grown on α-Al_2_O_3_(0001) exhibits stable adhesion to the substrate and possess a large surface area, we are interested in exploring a broad spectrum of potential applications. These applications may include sensing materials and primers in addition to adsorbents, which could be investigated in future research.

## Experimental

### Growth of SLGO on α-Al_2_O_3_(0001)

SLGO was grown by a method analogous to [35]. Before synthesizing SLGO, SLG was grown on an α-Al_2_O_3_(0001) substrate (size: 10 × 10 mm^2^, thickness: 430 µm). The substrate was annealed at 900 °C in open air in order to obtain an atomically flat surface. Then, the substrate was introduced into a vacuum furnace. The base pressure of the furnace was 6 × 10^−6^ Pa. After evacuating, the substrate was annealed up to 1000 °C for 1 h. For the graphene growth, a methanol vapor was used as a precursor. The SLG was grown by introducing 200 Pa methanol vapor for 30 min [18]. The single layer of graphene was inspected by the intensity analysis of XPS and the profiles of NIXSW for graphene/α-Al_2_O_3_(0001). After the SLG growth, the SLG in the sample was oxidized based on the modified Hummers method [24]. The procedure was as follows: The mixture of H_2_SO_4_ and KMnO_4_ was prepared by slowly adding KMnO_4_ (1.8 g) into concentrated H_2_SO_4_ (20 mL) and stirring in the beaker. SLG/α-Al_2_O_3_(0001) was dipped in the mixture for 30 s. The oxidized specimen was washed with purified water and dried under nitrogen gas blow.

### Cs adsorption

Prior to Cs adsorption, the SLGO surface was treated with aqueous solutions which were adjusted to pH 4, 7, and 9 prior to the adsorption of the Cs atoms. Solutions with pH values of 4 and 9 were adjusted with acetate and borate buffer solutions, respectively. Then the SLGO surface was dipped into a 1.0 mol/L CsCl solution with the same pH values. The solutions were kept at room temperature. Both pH adjustment and CsCl solutions were added dropwise on the SLGO surface. The volumes of the solutions and contact times were 1 µL and 5 min, respectively. After Cs adsorption, the CsCl solution was removed with a water rinse for 5 min.

### Characterization

The electronic structure of Cs-adsorbed SLGO was investigated by NEXAFS. The C and O K-edge NEXAFS measurements were carried out at the BL-8 station in the Ritsumeikan Synchrotron Radiation Center. The partial electron-yield method was employed to obtain the spectra. The amount of Cs adsorption was inspected by XPS. The energy scan for XPS was done with a VSW CLASS 100 hemispherical energy analyzer. An Al Kα (1486.6 eV) X-ray source (PSP TX400/2) was used for the excitation. In the XPS measurement, the CsCl solution was removed from the SLGO surface by water rinsing and then the Cs-adsorbed SLGO specimen was introduced in the XPS chamber kept at ultra-high vacuum. The surface morphology of SLGO was examined using atomic force microscopy (AFM, SII SAP300).

## Data Availability

All data that supports the findings of this study is available in the published article and/or the supporting information of this article.
